# New Cytotoxic Oxygenated Sterols from the Marine Bryozoan *Cryptosula pallasiana*

**DOI:** 10.3390/md9020162

**Published:** 2011-01-28

**Authors:** Xiang-Rong Tian, Hai-Feng Tang, Yu-Shan Li, Hou-Wen Lin, Xiao-Li Chen, Ning Ma, Min-Na Yao, Ping-Hu Zhang

**Affiliations:** 1 Department of Pharmacy, Xijing Hospital, Fourth Military Medical University, Xi’an 710032, Shannxi, China; Email: tianxangrong@163.com (X.-R.T.); 2 School of Traditional Chinese Medicines, Shenyang Pharmaceutical University, Shenyang 110016, Liaoning, China; 3 Department of Pharmacy, Changzheng Hospital, Second Military Medical University, Shanghai 200433, China; Email: franklin67@126.com (H.-W.L.); 4 Jiangsu Center for Drug Screening &amp; National Drug Screening Laboratory, China Pharmaceutical University, Nanjing 210009, Jiangsu, China; Email: 308694164@qq.com (P.-H.Z.)

**Keywords:** marine bryozoan, *Cryptosula pallasiana*, oxygenated sterols, cytotoxicity

## Abstract

Six new sterols (**1**-**6**), together with seven known sterols (**7**-**13**), were isolated from the CCl_4_ extract of the marine bryozoan *Cryptosula pallasiana*, four (**3**-**6**) of which have already been reported as synthetic sterols. This is the first time that these compounds (**3**-**6**) are reported as natural sterols. The structures of the new compounds were determined on the basis of the extensive spectroscopic analysis, including two-dimensional (2D) NMR and HR-ESI-MS data. Compounds **1**-**4**, **7** and **10**-**13** were evaluated for their cytotoxicity against HL-60 human myeloid leukemia cell line, and all of the evaluated compounds exhibited moderate cytotoxicity to HL-60 cells with a range of IC_50_ values from 14.73 to 22.11 µg/mL except for compounds **12** and **13**.

## 1. Introduction

Marine bryozoans are well known producers of bioactive secondary metabolites and important marine drug sources due to their remarkable antineoplastic activity [1]. Bryostatins isolated from the marine bryozoans *Bugula neritina* are a well known example [2]. Other bioactive secondary metabolities from marine bryozoans include alkaloids, sterols, as well as heteratom-containing compounds, which showed remarkable activities on tumor cell lines, such as murine lymphocytic leukemia P388, human myeloid leukemia HL-60, human leukemia U937, human hepatocellular liver carcinoma HepG2, *etc*. [3,4,5,6]. In our previous studies focused on *B. neritina*, a new antineoplastic macrolide, bryostatin 19, two ceramides and four cerebrosides, as well as a series of sterols were isolated from this bryozoan [5,6,7,8]. In the course of our ongoing investigations toward the isolation of biologically active secondary metabolites from marine bryozoans, *Cryptosula pallasiana* was investigated, another genus of marine bryozoans, collected from the coast of Huang Island in Qingdao City, Shandong Province of China. Herein, we report the isolation and structure identification of six new sterols (**1**-**6**) and seven known sterols (**7**-**13**), four (**3**-**6**) of which have already been reported as synthetic sterols [9,10,11]. This is the first time that these compounds (**3**-**6**) are reported as natural sterols. In addition, the cytotoxicity of the oxygenated sterols **1**-**4**, **7** and **10**-**13** against HL-60 human myeloid leukemia cell line is also described.

**Figure 1:**
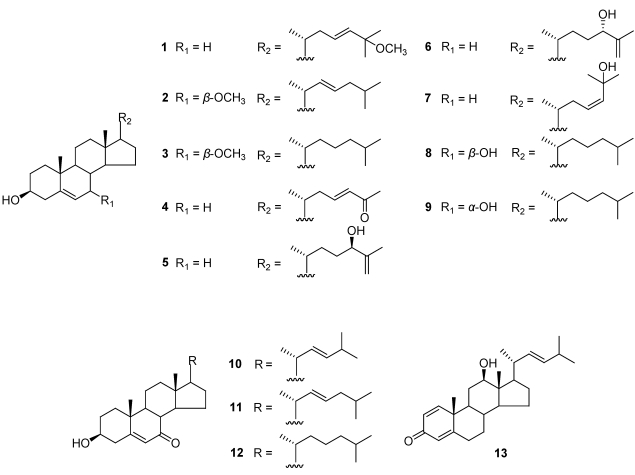
Chemical structures of compounds ** 1**-**13** from the marine bryozoan *Cryptosula pallasiana.*

## 2. Results and Discussion

The CCl_4_ extract (12.9 g) of the marine bryozoan *C. pallasiana* was fractionated by Sephadex LH-20 chromatography to afford three major fractions (Frs. A-C). Fr. A (5.66 g) was further subjected to column chromatography (CC) over reversed-phase silica gel column (RP-18) and normal silica gel column, respectively, and then further purified by reverse semi-preparation HPLC to yield compounds **1**-**13** (Figure 1).

**Table 1:** ^1^H NMR (500 MHz) and ^13^C NMR (125 MHz) data of compounds **1** and **2** in CDCl_3_^a^.

	**1**			**2**	
**Position**	***δ*_c_, mult.**	***δ*_H_,(int., mult., *J* in Hz)**		***δ*_c_, mult.**	***δ*_H_,(int., mult., *J* in Hz)**
1	37.4 t	*α*1.84 (1H, m), *β*1.14 (1H, m)		36.9 t	*α*1.82 (1H, m), *β*1.16 (1H, m)
2	31.8 t	*α* 1.83 (1H, m), *β*1.49 (1H, m)		31.6 t	*α*1.84 (1H, m), *β*1.51 (1H, m)
3	71.9 d	3.52 (1H, m)		71.6 d	3.61 (1H, m)
4	42.4 t	*α*2.28 (1H, m), *β*2.23 (1H, m)		42.5 t	*α*2.33 (1H, m), *β*2.29 (1H, m)
5	140.9 s	−		146.2 s	−
6	121.8 d	5.35 (1H, t, 2.8)		120.9 d	5.73 (1H, dd, 5.0, 1.7)
7	32.0 t	*α*1.48 (1H, m), *β*1.97 (1H, m)		74.1 d	3.27 (1H, m)
8	32.0 d	1.46 (1H, m)		37.3 d	1.50 (1H, m)
9	50.2 d	0.93 (1H, m)		42.9 d	1.31 (1H, m)
10	36.6 s	−		37.6 s	−
11	21.2 t	*α*1.00 (1H, m), *β*1.47 (1H, m)		20.9 t	*α*1.02 (1H, m), *β*1.49 (1H, m)
12	39.9 t	*α*1.15 (1H, m), *β*1.99 (1H, m)		39.1 t	*α*1.19 (1H, m), *β*1.94 (1H, dt, 12.6, 3.6)
13	42.5 s	−		42.2 s	−
14	56.9 d	0.98 (1H, m)		49.3 d	1.51 (1H, m)
15	24.5 t	*α*1.59 (1H, m), *β*1.08 (1H, m)		24.4 t	*α*1.59 (1H, m), *β*1.07 (1H, m)
16	28.4 t	*α*1.84 (1H, m), *β*1.28 (1H, m)		28.8 t	*α*1.74 (1H, m), *β*1.26 (1H, m)
17	56.0 d	1.10 (1H, m)		55.8 d	1.21 (1H, m)
18	12.1 q	0.69 (3H, s)		11.8 q	0.67 (3H, s)
19	19.5 q	1.00 (3H, s)		18.4 q	0.98 (3H, s)
20	36.2 d	1.47 (1H, m)		40.3 d	2.05 (1H, m)
21	18.9 q	0.91 (3H, d, 6.6)		21.0 q	1.01 (3H, d, 6.6)
22	39.3 t	a 2.17 (1H, m), b 1.78 (1H, m)		138.4 d	5.22 (1H, dd, 15.2, 8.1)
23	128.8 d	5.50 (1H, m)		126.3 d	5.27 (1H, m)
24	136.8 d	5.38 (1H, d, 15.8)		42.1 t	1.83 (2H, m)
25	75.0 s	−		28.7 d	1.58 (1H, m)
26	25.9 q	1.25 (3H, s)		22.4 q	0.86 (3H, d, 1.9)
27	26.3 q	1.25 (3H, s)		22.5 q	0.83 (3H, d, 1.9)
7−OCH_3_	−	−		56.9 q	3.35 (3H, s)
25−OCH_3_	50.4 q	3.15 (3H, s)		−	−

^a^ Assignments aided by the DEPT, COSY, TOCSY, HSQC, HMBC, and NOESY experiments.

Compound **1** was obtained as a white amorphous powder and was positive to Liebermann-Burchard test. The positive ion mode HR-ESI-MS spectrum showed a pseudomolecular ion peak at *m/z* 437.3398 [M + Na]^+^ (C_28_H_46_O_2_Na, calculated for 437.3396), which, together with the molecular ion peak at *m/z* 414 [M]^+^ in the positive ion mode EI-MS, enabled the determination of the molecular formula C_28_H_46_O_2_, with the help of NMR data (Table 1).

**Figure 2:**
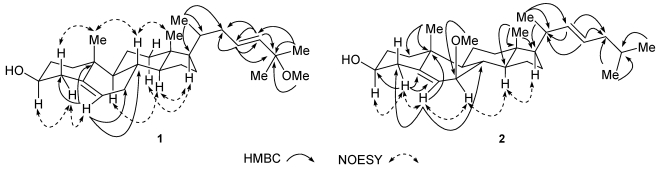
Key HMBC and NOESY correlations of compounds **1** and **2**.

An extensive examination of ^1^H NMR and ^13^C NMR spectra data, to draw assistance from the data of ^1^H-^1^H COSY, HSQC and HMBC spectra, allowed the establishment of a sterol skeleton with a 5(6)-double bond (*δ*_C_ 140.9 and 121.8), which was consistent with the literature [12]. The ^1^H NMR spectrum showed five methyl resonance signals at *δ*_H_0.69 (3H, s), 0.91 (3H, d, *J* = 6.6 Hz), 1.00 (3H, s), and 1.25 (6H, s), which were ascribed to the methyl groups 18, 21, 19 and 26/27, respectively. The resonances at *δ*_H_3.52 (1H, m) and 5.35 (1H, t, *J* = 2.9 Hz) assigned for H-3 and the olefinic proton H-6, respectively, were indicative for Δ^5^ mon-hydroxylated steroidal nucleus, which was confirmed by HMBC correlations from H-6 to C-4 (*δ*_C_ 42.4), C-7 (*δ*_C_ 32.0), C-8 (*δ*_C_ 32.0) and C-10 (*δ*_C_ 36.6) (Figure 2), as well as ^1^H-^1^H COSY correlations from H-3 to H_2_-2 and H_2_-4. The partial structure of C-17 side chain in compound **1** was established based on EI-MS, 1D and 2D NMR data. The double bond of C-23 (*δ*_C_128.8) and C-24 (*δ*_C_ 136.8) was confirmed by the cross peaks of H-23 (*δ*_H_5.50, m) to C-22 (*δ*_C_ 39.3), C-24 and C-25 (*δ*_C_ 75.0); H-24 (*δ*_H_5.38, d, *J* = 15.8 Hz) to C-22 and C-23 in the HMBC spectrum, and was determined to be *trans*-disubstituted due to the large coupling constant (*J*_23,24_ = 15.8 Hz). The methoxy group (*δ*_H_3.15 (3H, s), *δ*_C_ 50.4) at C-25 position was confirmed by the HMBC spectrum data (Figure 2) and fragment ion at *m/z* 382 [M *−*OCH_3_ *−*H]^+^ in the EI-MS spectrum of **1**. Based on the above analysis, the plane structure of **1** was determined (Figure 1). 

In the NOESY experiment, both H-3 and H-6 correlated with H-4*α* (*δ*_H_2.28, m), indicating the *β*-orientation of the hydroxyl group on C-3 (Figure 2), which was confirmed by the chemical shift of C-3 (*δ*_C_71.9 > 70.0) [13]. After mapping all of the signals for each moiety by careful inspection of the 1D and 2D NMR spectra, compound **1** was unambiguously assigned as (23*E*)-25-methoxy-cholesta-5,23-dien-3*β*-ol. Sterol **1**, not reported previously in the literature, is a new sterol with *trans*-double bonds between C-23 and C-24, together with a methoxy group at C-25 in the side chain.

Compound **2** was isolated as an isomer of **1**, due to the same formula of C_28_H_46_O_2_ from EI-MS (*m/z* 414 [M]^+^) and HR-ESI-MS (*m/z* 437.3394 [M + Na]^+^ (C_28_H_46_O_2_Na, calculated for 437.3396)), with the help of 1D NMR spectral data. Fragment at *m/z* 382 [M *−*OCH_3_ *−*H]^+^ due to loss of a methoxy group, as well as the two ion fragments at *m/z* 271 [M *−*C_8_H_15_ *−*OCH_3_ *−*H]^+^ and 253 [M *−*C_8_H_15_ *−*OCH_3_ *−*H *−*H_2_O]^+^, suggested the presence of the mono-hydroxylated and mono-methoxylated steroid with a mono-unsaturated side chain. The presence of a strong peak in the EI-MS at *m/z* 111 confirmed the presence of the C_8_H_15_ side chain. The partial structure of a Δ^22^ mono-unsaturated side chain was established from the HMBC spectrum of **2** (Figure 2), which showed correlations of H_3_-27/C-25, H_3_-26/C-25, H_3_-26/C-24, H-23/C-24, H-23/C-20, H-22/C-23, H-22/C-20, H_3_-21/C-17, H_3_-21/C-20 and H_3_-21/C-22. The position of the methoxy group was assigned as C-7 (*δ*_C _74.1) due to a strong broad singlet at *δ*_H_3.35 (3H, s) correlated to C-7 in the HMBC spectrum, and the partial structure of the steroidal nucleus was confirmed by the observation of the HMBC cross peaks from H_2_-4 to C-3, C-5 and C-6; H-6 to C-4, C-8 and C-10; H_3_-18 to C-13, C-14 and C-17, and H_3_-19 to C-1 and C-10 (Figure 2). The configuration of the double bond between C-22 and C-23 was determined to be *trans*-disubstituted due to the large coupling constant (*J*_22,23_ = 15.2 Hz) between H-22 and H-23. The *β*-orientation of the hydroxyl group on C-3 was deduced also from the NOESY correlations of H-3/H-4*α* and H-4*α*/H-6, and the *β*-orientation of the methoxy group on C-7 was deduced by observation of correlations of H-7*α*/H-6 and H-7*α*/H-14 in the NOESY experiment (Figure 2). Accordingly, compound **2** can be defined as (22*E*)-7*β*-methoxy-cholesta-5,22-dien-3*β*-ol.

Compound **3** was isolated as a white amorphous powder and was also positive to the Libermann-Burchard test. Its molecular formula was established as C_28_H_48_O_2_ by observation of the molecular ion peak at *m/z* 439.3550 [M + Na]^+^ (C_28_H_48_O_2_Na, calculated for 439.3552) in the HR-ESI-MS spectrumand ion peak at *m/z* 416 [M]^+^ in the EI-MS spectrum. Comparison of the ^1^H NMR and ^13^C NMR spectra of **3** with those of **2** revealed that they shared the same 3*β*-hydroxy, 7*β*-methoxy Δ^5^-steroid nucleus but differed in the side chain. Compound **3** was finally assigned as 7*β*-methoxy-cholest-5-en-3*β*-ol due to the missing *trans*-double bonds between C-22 and C-23 in the ^13^C NMR spectrum by comparison with **2**. Compound **3** has been reported as a synthetic sterol with effective inhibition of cholesterol acyltransferase (ACAT) [9] and is reported here as a natural product for the first time.

Compound **4** was obtained as a white amorphous powder and was positive to Liebermann-Burchard test. The HR-ESI-MS spectrum showed the molecular ion peak at *m/z* 407.2928 [M + Na]^+^ (C_26_H_40_O_2_Na, calculated for 407.2926) and EI-MS spectrum showed the molecular ion peak at *m/z* 384 [M]^+ ^corresponding to the molecular formula C_26_H_40_O_2_, with the help of NMR data. Compound **4** was assumed to have the same typical nucleus of 3*β*-hydroxy Δ^5^-steroid by comparing the 1D NMR data with those of **1**, but differed in the side chain. The protons of *trans*-olefinic bonds in the side chain appeared at *δ*_H_6.07 (1H, d, *J* = 15.5 Hz) and 6.78 (1H, m). The HSQC spectral data indicated that the proton H-24 (*δ*_H_6.07) was connected to the carbon at *δ*_C_132.8 (C-24) and H-23 (*δ*_H_6.78) was connected to the carbon at *δ*_C_147.6 (C-23), while the protons H_2_-22 (*δ*_H_2.34, 1.98) were connected to the carbon at *δ*_C_39.5 (C-22). The correlation of H-23 with H_2_-22 and H-24 in the ^1^H-^1^H COSY spectrum indicated that the double bonds were in C-23 and C-24. This was also supported by the key cross-peaks H-24 with C-22 and H-23 with C-22 in the HMBC experiment. Similarly, the downfield singlet methyl protons at *δ*_H_ 2.25 (H_3_-26) exhibited HMBC correlations with C-25 (*δ*_C_198.7) and C-24, and upfield methyl proton signals at *δ*_H_0.95 (H_3_-21) with C-17 (*δ*_C_55.9), C-20 (*δ*_C_36.0) and C-22, suggesting the remnant connectivities of the side chain in compound **4**. Accordingly, **4** was finally assigned as (23*E*)-3*β*-hydroxy-27-norcholesta-5,23-dien-25-one, which had been obtained by synthesis [10], but was reported as a natural product for the first time.

Another two stereoisomeric sterols, **5** and **6**, were isolated as white amorphous powder, and were analyzed to share the same molecular formula of C_27_H_44_O_2 _using EI-MS (*m/z* 400 [M]^+^) and HR-ESI-MS data. The ^1^H NMR and^ 13^C NMR data of **5** and **6** agreed with those of cholesta-5,25-diene-3*β*,24*ξ*-diol from red alga *Galaxaura marginata* [14]. Although, the configuration at C-24 was hard to determine due to the small difference in the chemical shift of C-24 in the ^13^C NMR spectrum between *S* and *R* epimers (Table 2), compound **5** was finally assigned as 24(*R*)-cholesta-5,25-diene-3*β*,24-diol due to no correlation between H-24 and H-20*β* in the NOESY experiment, whereas, the presence of H-24 correlated with H-20*β* in **6** confirmed the correct configuration of C-24 in **5**. Furthermore, the results were confirmed by the ^1^H NMR data of **5** and **6**, consistent with the same synthetic sterols reported earlier [11]. Accordingly, **6** was assigned to be 24(*S*)-cholesta-5,25-diene-3*β*,24-diol. **5** and **6** as stereoisomeric sterols were isolated from a natural origin for the first time.

**Table 2:** ^13^C NMR data of compounds **3**-**6** (CDCl_3_, 125 MHz) ^a^.

	**3**		**4**		**5**		**6**
**Position**	***δ*_C_, mult.**		***δ*_C_, mult.**		***δ*_C_, mult.**		***δ*_C_, mult.**
1	36.9 t		37.4 t		37.4 t		37.4 t
2	31.6 t		31.8 t		31.8 t		31.8 t
3	71.6 d		71.9 d		71.9 d		71.9 d
4	42.5 t		42.5 t		42.4 t		42.4 t
5	146.2 s		140.9 s		140.9 s		140.9 s
6	120.9 d		121.8 d		121.8 d		121.9 d
7	74.1 t		32.0 t		32.0 t		32.1 t
8	37.3 d		32.1 d		32.0 d		32.1 d
9	42.9 d		50.2 d		50.3 d		50.3 d
10	37.6 s		36.6 s		36.7 s		36.7 s
11	20.9 t		21.2 t		21.2 t		21.2 t
12	39.2 t		39.8 t		39.9 t		39.9 t
13	42.2 s		42.6 s		42.5 s		42.5 s
14	49.2 d		56.8 d		56.9 d		56.9 d
15	24.4 t		24.4 t		24.4 t		24.4 t
16	28.8 t		28.5 t		28.3 t		28.4 t
17	55.9 d		55.9 d		56.0 d		56.0 d
18	11.6 q		12.0 q		12.0 q		12.0 q
19	18.4 q		19.5 q		19.8 q		19.6 q
20	36.0 d		36.0 d		35.7 d		35.7 d
21	18.9 q		19.2 q		18.9 q		18.9 q
22	36.3 t		39.5 t		31.8 t		31.8 t
23	23.9 t		147.6 d		31.4 t		31.5 t
24	39.7 t		132.8 d		76.9 d		76.5 d
25	28.2 d		198.7 s		147.6 d		147.9 d
26	22.7 q		27.1 q		111.5 t		111.0 t
27	23.0 q		−		17.4 q		17.8 q
7−OCH_3_	56.9 q		−		−		−

^a^ Assignments aided by the DEPT, COSY, TOCSY, HSQC, HMBC, and NOESY experiments.

Comparing their MS and NMR data with those reported in the literature, the known sterols were identified as (23*Z*)-cholesta-5,23-diene-3*β*,25-diol (**7**) [15], cholest-5-ene-3*β*,7*β*-diol (**8**) [16], cholest-5-ene-3*β*,7*α*-diol (**9**) [16], (22*E*)-3*β*-hydroxy-24-norcholesta-5,22-dien-7-one (**10**) [16], (22*E*)-3*β*-hydroxycholesta-5,22-dien-7-one (**11**) [16], 3*β*-hydroxycholest-5-en-7-one (**12**) [16] and (4*E*,22*E*)-12*β*-hydroxy-24-norcholesta-1,4,22-trien-3-one (**13**) [17], respectively. The *cis*-double bonds between C-23 and C-24, together with an oxygenated hydroxyl group at C-25 in the side chain of **7** is scarce in natural sterols. Sterols **8**-**12** were previously isolated from two marine sponges *Cliona copiosa* [16] and *Stelodoryx chlorophylla* [18], and sterols **8**-**9** were also isolated from a soft coral *Dendronephthya gigantean* [19] and a marine bryozoan *Biflustra grandicella* [20]. Compound **13** was a highly functionalized C_26_ steroid Δ^1,4^-dien-3-one with 12*β* oxygen function, which is a rare structural feature among sterols and has been isolated from soft coral *Gersemia rubiformis* [17]. However, all of the known sterols were isolated for the first time from this species.

Compounds **1**-**4**, **7** and **10**-**13** were evaluated for their cytotoxicity against HL-60 human myeloid leukemia cells *in vitro*, using a MTT assay method. The results of their cytotoxicity are shown in Table 3. Although **12** and **13** did not show any apparent cytotoxicity, sterols **1**-**4**, **7**, **10**  and **11** displayed moderate cytotoxicity to HL-60 cells with IC_50_ values of 17.91, 21.30, 22.11, 15.05, 18.28, 15.12 and 14.73 μg/mL, respectively. It appears that the cytotoxicity against HL-60 human myeloid leukemia cells of these sterols has a correlation with their structure.

**Table 3:** Cytotoxic activities of compounds **1**-**4**, **7** and **10**-**1** **3** on HL-60 tumor cells ^a^.

**Compound**	**IC_50_ (µg/mL)**		**Compound**	**IC_50_ (µg/mL)**
**1**	17.91		**10**	15.12
**2**	21.30		**11**	14.73
**3**	22.11		**12**	NA
**4**	15.05		**13**	NA
**7**	18.28		Adriamycin	2.50

^a ^IC_50_: 50% inhibitory concentration, HL-60: human myeloid leukemia cell line, NA: no activity.

The present chemical study of the marine bryozoan *C. pallasiana* resulted in the isolation and characterization of six new sterols (**1**-**6**) and seven known sterols (**7**-**13**), four (**3**-**6**) of which have already been reported as synthetic sterols [9,10,11]. This is the first time that they (**3**-**6**) are reported as natural sterols. The structures of these new compounds are notable for the following viewpoints of natural product chemistry. Sterol **1**,not reported previously in the literature, is characterized by an oxygenated methoxy group at C-25 in the side chain. In the nucleus of **2** and **3**, the 7*β* methoxy group is a rare feature and first encountered among natural sterols. Compound **4** is specific in carbonylation at C-25 accompanied by a loss of a methyl group in the side chain. Sterols **5** and **6** are stereoisomeric with the C-24 with hydroxyl group in the side chain, which are reported as a natural source for the first time. 

## 3. Experimental Section

### 3.1. General Experimental Procedures

Optical rotations were measured on a Perkin-Elmer 343 polarimeter. 1D and 2D NMR spectra experiments were measured in CDCl_3_ on a Bruker AVANCE-500 spectrometer, with TMS as an internal standard. Chemical shifts (*δ*) were expressed in ppm and coupling constants in Hz. EI-MS spectra were obtained on a MAT212 mass spectrometer; ESI-MS and HR-ESI-MS spectra were taken on a Micromass Quattro mass spectrometer. Separation and purification were performed by CC on silica gel H (10−40 μm, Qingdao Marine Chemical Inc., Qingdao, China), Sephadex LH-20 (Pharmacia Inc., New Jersey, USA), reversed-phase Si gel (Lichroprep RP-18, 40-63 μm, Merck Inc., Darmstadt, Germany). HPLC was carried out on a Dionex P680 liquid chromatograph equipped with a UV 170 UV/Vis detector at 206 nm using a YMC-Pack R﹠D ODS-A column (250 ° 20 mm i.d., 5 μm, YMC, Kyoto, Japan) for semi-preparation and a Thermo ODS-2 column (250 ° 4.6 mm i.d., 5 μm, Thermo Hypersi-Keystone Inc., Bellefonte, U.S.A.) for analysis. TLC detection was achieved by spraying the silica gel plates (Qingdao Marine Chemical Inc., Qingdao, China) with 20% H_2_SO_4_ followed by heating.

### 3.2. Animal Material

The samples of marine bryozoan *Cryptosula pallasiana* were collected in March 2009 from Huang Island, Qingdao City, Shandong Province of China, and were identified by one of the authors (Prof. H.-W. Lin). A voucher specimen (No: QD-0903-1) was deposited in Marine Laboratory, Changzheng Hospital, Second Military Medical University.

### 3.3. Extraction and Isolation

Fresh samples of *Cryptosula pallasiana* (about 20 kg) were extracted with 95% EtOH at ambient temperature. The concentrated aqueous solution was extracted with EtOAc. Then, the extract was partitioned between 90% aqueous MeOH and petroleum ether. The MeOH solution was adjusted to 80% aqueous MeOH and extracted with CCl_4_. The CCl_4 _fraction (12.9 g) was subjected to column chromatography (CC) on Sephadex LH-20 with CHCl_3_/MeOH (1:1) as eluting solvent to afford three fractions (Frs. A−C) based on TLC analysis (developed by petroleum ether/EtOH, 5:1). Fr. A (5.66 g) was subjected to CC on reversed-phase silica gel column eluting with MeOH/H_2_O (80:20 to 100:0) gradient to give two major fractions A_1_ (2.44 g) and A_2 _(3.18 g). Fr. A_2_ was submitted to CC over silica gel eluting with petroleum ether/EtOAc (15:1, 10:1, 5:1, 1:1) gradient to give 12 major fractions (Frs. A_2_-1−A_2_-12). Fr. A_2_-7 (238.9 mg) was eluted with CHCl_3_/MeOH (1:1) on Sephadex LH-20 and then further purified by semi-preparative HPLC to afford **1** (6.0 mg, *t*_R_ = 97.7 min), **5** (12.0 mg, *t*_R_ = 34.0 min), **6** (11.2 mg, *t*_R_ = 35.6 min) and **7** (11.4 mg, *t*_R_ = 38.4 min), using MeOH/H_2_O (87:13) as the mobile phase at a flow rate of 8.0 mL/min. Fr. A_2_-6 was purified by semi-preparative HPLC (MeOH/H_2_O 90:10, flow rate of 8.0 mL/min) to yield **2** (3.5 mg, *t*_R_ = 40.7 min), **3** (10.5 mg, *t*_R_ = 50.5 min) and **4** (2.0 mg, *t*_R_ = 16.8 min). Fr. A_2_-9 (577.0 mg) was firstly purified by semi-preparative HPLC (MeOH/H_2_O 90:10, flow rate of 8.0 mL/min) to give 12 fractions (Frs. A_2_-9-1−A_2_-9-12), and then the Fr. A_2_-9-12 (47.4 mg) was further purified by analytic HPLC to afford **8** (14.8 mg, *t*_R_ = 32.1 min) and **9** (18.2 mg, *t*_R_ = 35.4 min), using MeOH/H_2_O (87:13) as the mobile phase at a flow rate of 1.0 mL/min. Fr. A_2_-8 (147.5 mg) was eluted with CHCl_3_/MeOH (1:1) on Sephadex LH-20 and then further purified by semi-preparative HPLC to give **10** (3.4 mg, *t*_R_ = 71.8 min), **11** (4.1 mg, *t*_R_ = 98.3 min) and **12** (8.9 mg, *t*_R_ = 130.5 min), **13** (4.0 mg, *t*_R_ = 36.5 min), using MeOH/H_2_O (90:10) as the mobile phase at a flow rate of 8.0 mL/min. 

#### 3.3.1. Liebermann-Burchard Test

Each sample (1-2 mg) was dissolved in a mixture of 2 mL CHCl_3_ and anhydrous acetic acid (1:1), then a few drops of concentrated sulfuric acid was added and mixed cautiously. The appearance of a green color indicated the presence of sterol.

#### 3.3.2. (23*E*)-25-Methoxy-cholesta-5,23-dien-3β-ol (**1**)

White amorphous powder; [α]22D − 40.7° (c 0.05, CHCl_3_); ^1^H NMR and ^13^C NMR data, see Table 1; EI-MS m/z: 414 [M]^+^ (16), 399 [M − CH_3_]^+^ (100), 382 [M − OCH_3 _− H]^+^ (26), 367 (18), 349 (11), 301 [M − C_6_H_10_OCH_3_]^+ ^(25), 300 (30), 283 [M − C_6_H_10_OCH_3 _− H_2_O]^+^ (37), 271 (46), 241 (12), 227 (10), 215 (24), 183 (19), 159 (27), 133 (31), 113 [C_6_H_10_OCH_3_]^+^ (28), 99 (47), 85 (61), 55 (42); HR-ESI-MS (positive) m/z: 437.3398 [M + Na]^+^ (C_28_H_46_O_2_Na, calcd. for 437.3396).

#### 3.3.3. (22*E*)-7β-Methoxy-cholesta-5,22-dien-3β-ol (**2**)

White amorphous powder; [α]22D − 67.5° (c 0.05, CHCl_3_); ^1^H NMR and ^13^C NMR data, see Table 1; EI-MS m/z: 414 [M]^+^ (38), 396 [M − H_2_O]^+^ (42), 382 [M − OCH_3 _− H]^+^ (100), 367 (8), 349 (12), 303 [M − C_8_H_15_]^+^ (10), 298 (15), 271 [M − C_8_H_15 _− OCH_3 _− H]^+^ (22), 253 (21), 211 (13), 197 (9), 175 (14), 159 (17), 145 (19), 135 (17), 119 (16), 111 [C_8_H_15_]^+^ (22), 69 (30), 55 (37); HR-ESI-MS (positive) m/z: 437.3394 [M + Na]^+^ (C_28_H_46_O_2_Na, calcd. for 437.3396).

#### 3.3.4. 7β-Methoxy-cholest-5-en-3β-ol (**3**)

White amorphous powder; [α]22D − 72.4° (c 0.10, CHCl_3_); ^1^H NMR (500 MHz, CDCl_3_) δ: 0.66 (3H, s, H_3_-18), 0.86 (3H, d, J = 2.3 Hz, H_3_-27), 0.87 (3H, d, J = 2.3 Hz, H_3_-26), 0.91 (3H, d, J = 6.5 Hz, H_3_-21), 0.98 (3H, s, H_3_-19), 2.34 (1H, m, H_α_-4), 2.29 (1H, m, H_β_-4), 3.29 (1H, t, J = 3.3 Hz, H-7), 3.36 (3H, s, H_3_-7−OCH_3_), 3.62 (1H, m, H-3), 5.73 (1H, dd, J = 4.9, 1.6 Hz, H-6); ^13^C NMR data, see Table 2; EI-MS m/z: 416 [M]^+^ (26), 398 [M − H_2_O]^+^ (37), 384 [M − OCH_3 _− H]^+^ (100), 369 (11), 351 (12), 271 [M − C_8_H_17 _− OCH_3 _− H]^+^ (7), 253 (5), 213 (6), 211 (7), 185 (5), 175 (9),159 (12), 145 (12), 119 (12), 95 (15), 81 (14), 69 (11), 55 (15); HR-ESI-MS (positive) m/z: 439.3550 [M + Na]^+^ (C_28_H_48_O_2_Na, calcd. 439.3552).

#### 3.3.5. (23*E*)-3β-Hydroxy-27-norcholesta-5,23-dien-25-one (**4**)

White amorphous powder; [α]22D − 46.7° (c 0.02, CHCl_3_); ^1^H NMR (500 MHz, CDCl_3_) δ: 0.70 (3H, s, H_3_-18), 0.95 (3H, d, J = 6.6 Hz, H_3_-21), 1.01 (3H, s, H_3_-19), 2.25 (3H, s, H_3_-26), 3.52 (1H, m, H-3), 5.35 (1H, t, J = 2.8 Hz, H-6), 6.07 (1H, d, J = 15.5 Hz, H-24 ), 6.78 (1H, m, H-23); ^13^C NMR data, see Table 2; EI-MS m/z: 384 [M]^+^ (100), 366 [M − H_2_O]^+^ (66), 351 [M − CH_3 _− H_2_O]^+^ (40), 324 (9), 299 (29), 283 (32), 273 [M − Side Chain]^+^ (41), 255 [M − H_2_O − Side Chain]^+^ (23), 213 (45), 199 (19), 189 (29), 173 (24), 159 (48), 145 (54), 133 (44), 119 (46), 107 (62), 95 (52), 81 (54), 67 (38); HR-ESI-MS (positive) m/z: 407.2928 [M + Na]^+^ (C_26_H_40_O_2_Na, calcd. for 407.2926)

#### 3.3.6. 24(R)-Cholesta-5,25-diene-3β,24-diol (**5**)

White amorphous powder; [α]22D − 19.4° (c 0.10, CHCl_3_); ^1^H NMR (500 MHz, CDCl_3_) δ: 0.68 (3H, s, H_3_-18), 0.93 (3H, d, J = 6.6 Hz, H_3_-21), 1.00 (3H, s, H_3_-19), 1.72 (3H, s, H_3_-27), 3.52 (1H, m, H-3), 4.00 (1H, t, J = 6.6 Hz, H-24), 4.83 (1H, t, J = 1.4 Hz, H_a_-26), 4.92 (1H, s, H_b_-26), 5.35 (1H, d, J = 5.2 Hz, H-6); ^13^C NMR data, see Table 2; EI-MS m/z: 400 [M]^+^ (41), 382 [M − H_2_O]^+^ (56), 367 [M − CH_3 _− H_2_O]^+^ (33), 349 (25), 340 (10), 328 (13), 315 (22), 300 [M − C_6_H_10 _− H_2_O]^+^ (27), 271 [M − C_8_H_15 _− H_2_O]^+^ (100), 255 (36), 243 (17), 229 (31), 213 (46), 199 (23), 187 (26), 173 (30), 161 (49), 145 (52), 133 (46), 119 (46), 107 (58), 95 (56), 81 (60), 71 (65), 55 (63); HR-ESI-MS (positive) m/z: 423.3241 [M + Na]^+^ (C_27_H_44_O_2_Na, calcd. for 423.3239).

#### 3.3.7. 24(S)-Cholesta-5,25-diene-3β,24-diol (**6**)

White amorphous powder, [α]22D − 27.9° (c 0.10, CHCl_3_); ^1^H NMR (500 MHz, CDCl_3_) δ: 0.68 (3H, s, H_3_-18), 0.93 (3H, d, J = 6.6 Hz, H_3_-21), 1.00 (3H, s, H_3_-19), 1.72 (3H, s, H_3_-27), 3.52 (1H, m, H-3), 4.00 (1H, t, J = 6.4 Hz, H-24), 4.83 (1H, t, J = 1.4 Hz, H_a_-26), 4.93 (1H, s, H_b_-26), 5.35 (1H, d, J = 5.3 Hz, H-6); ^13^C NMR data, see Table 2; EI-MS m/z: 400 [M]^+^ (41), other ion fragments identical with **5**; HR-ESI-MS (positive) m/z: 423.3237 [M + Na]^+^ (C_27_H_44_O_2_Na, calcd. for 423.3239).

### 3.4. MTT Cytotoxicity Assays

The cytotoxicity of compounds **1**-**4**, **7** and **10**-**1** **3** were evaluated against HL-60 cancer cell line by microculture tetrazolium (MTT) assay [21]. The cells were obtained from American Type Culture Collection (ATCC), and maintained in RPMI 1640 medium (Gibco, Invitrogen Co., USA) containing 10% fetal bovine serum (Gibco, Invitrogen Co., USA) supplemented with 100 U/mL penicillin, and 100 U/mL streptomycin. The leukemia cells were washed and re-suspended in the above medium to 1 × 10^5^ cells/mL. 2 mL of this cell suspension was placed into 96-well microculture plates and allowed to adhere in 5% CO_2_/air for 24 h at 37 °C before drug addition. 20 µL of DMSO solution containing the sample was added to give the various concentrations in triplicate for 72 h, with adriamycin (Sigma) as positive control. After the incubation, 20 µL of MTT solution (5 mg/mL) was added to each well, and the incubation continued for 4 h at 37 °C. Then, 150 µL of DMSO solution was added to each well, and the formazan crystals in each well were dissolved by stirring with a pipette. The optical density (OD) was read on a plate reader on an ELISA reader (MK3, USA) at a wavelength of 570 nm. Each assay was done in triplicate, and inhibition was expressed as IC_50_ value, which stands for inhibition of cell growth by 50%.

## Figures and Tables

**S1:**
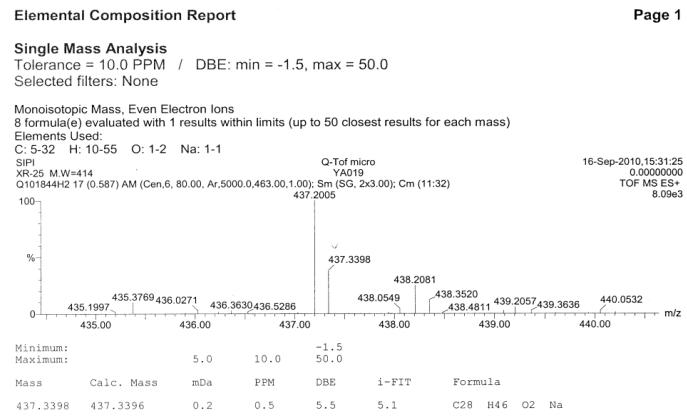
HR-ESI-MS of compound **1**

**S2:**
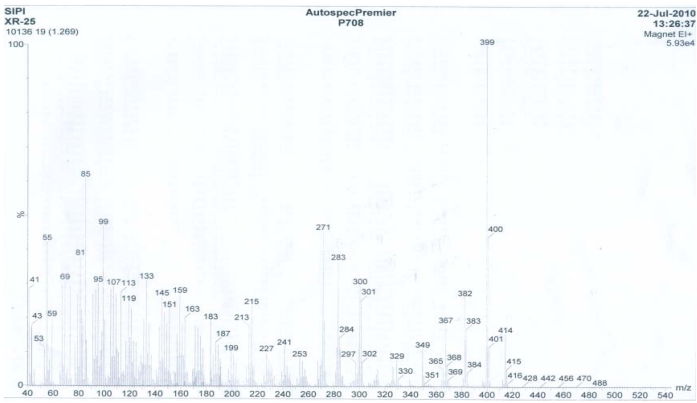
EI-MS of compound **1**

**S3:**
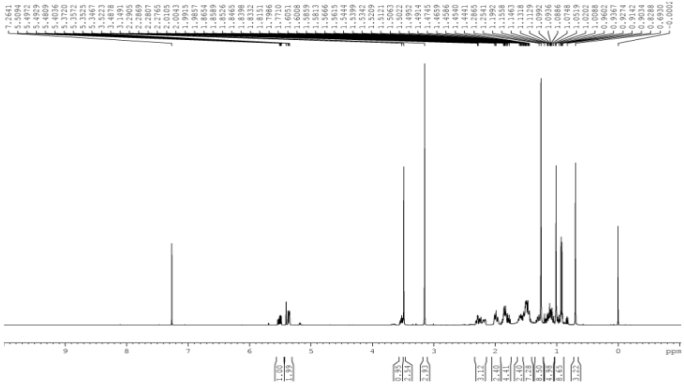
^1^H NMR spectrum (CDCl_3_, 500 MHz) of compound **1**

**S4:**
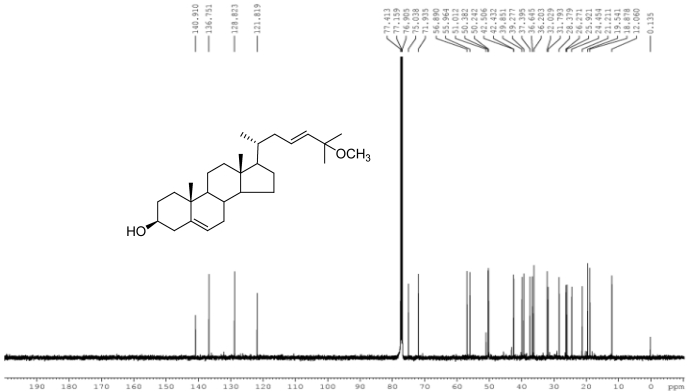
^13^C NMR spectrum (CDCl_3_, 125 MHz) of compound **1**

**S5:**
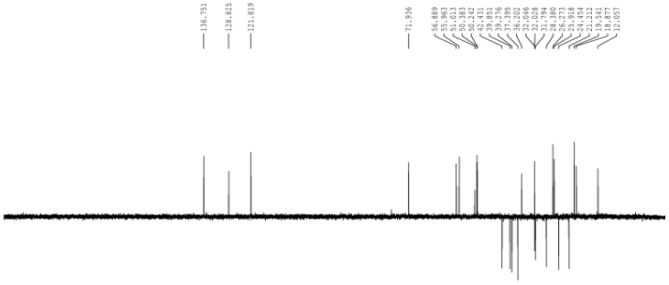
DEPT spectrum of compound **1**

**S6:**
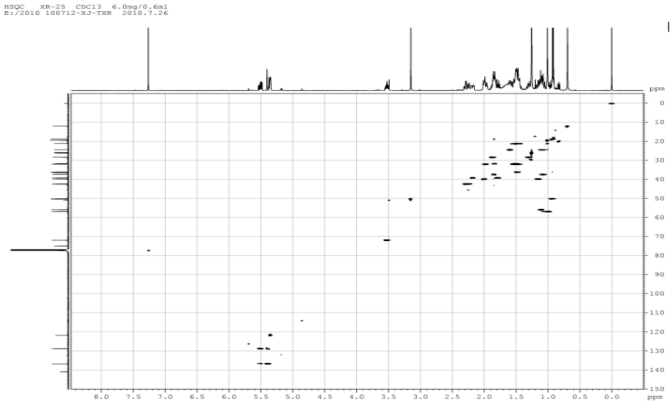
HSQC spectrum of compound **1**

**S7:**
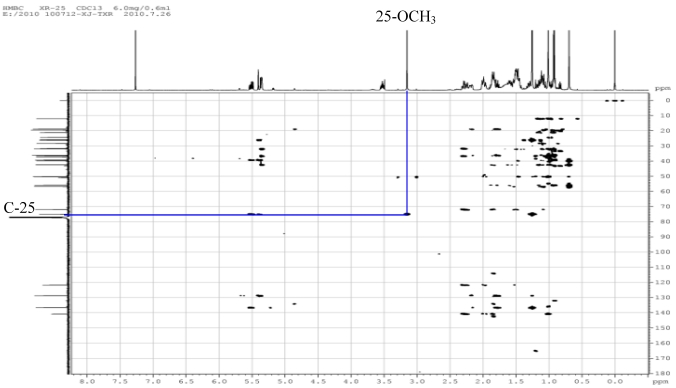
COSY spectrum of compound **1**

**S8:**
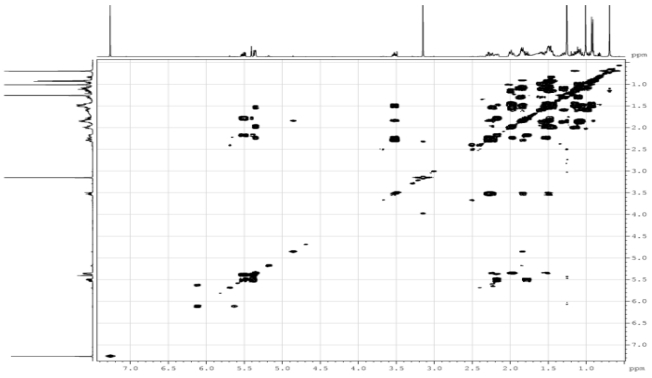
HMBC spectrum of compound **1**

**S9:**
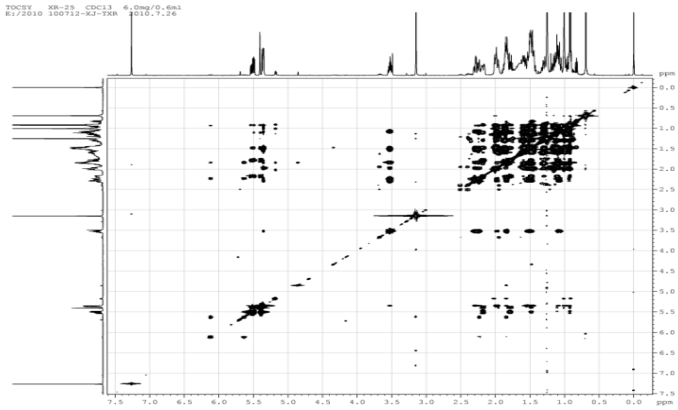
TOCSY spectrum of compound **1**

**S10:**
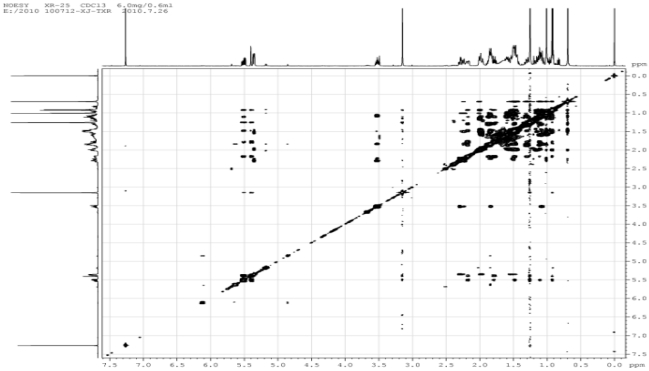
NOESY spectrum of compound **1**

**S11:**
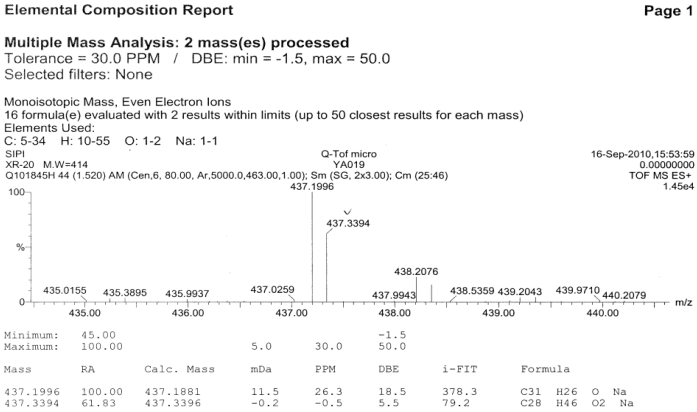
HR-ESI-MS of compound **2**

**S12:**
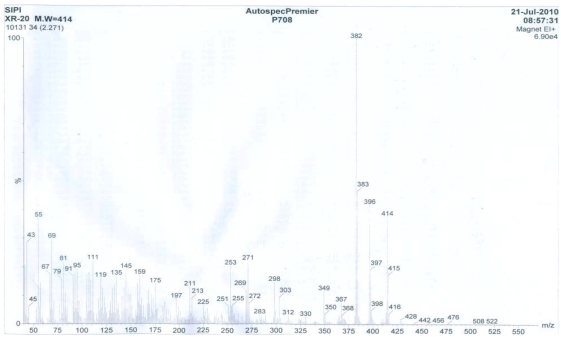
EI-MS of compound **2**

**S13:**
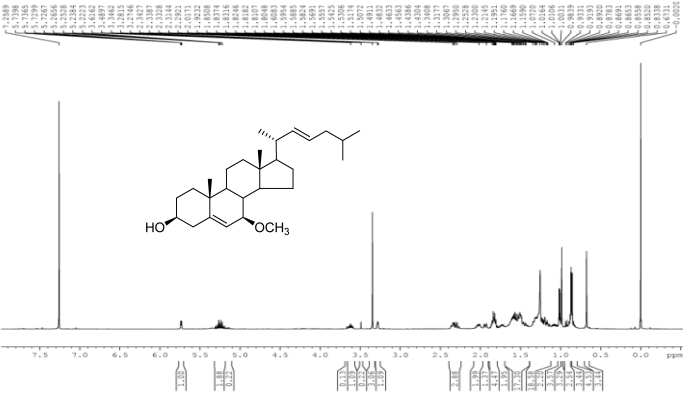
^1^H NMR spectrum (CDCl_3_, 500 MHz) of compound **2**

**S14:**
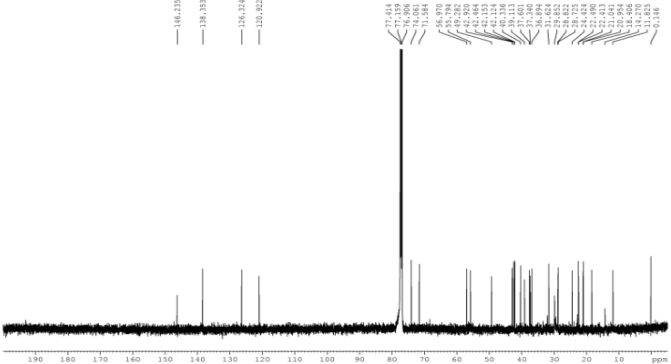
^13^C NMR spectrum (CDCl_3_, 125 MHz) of compound **2**

**S15:**
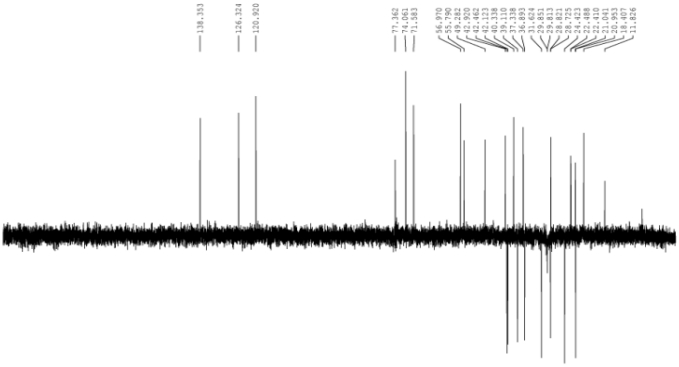
DEPT spectrum of compound **2**

**S16:**
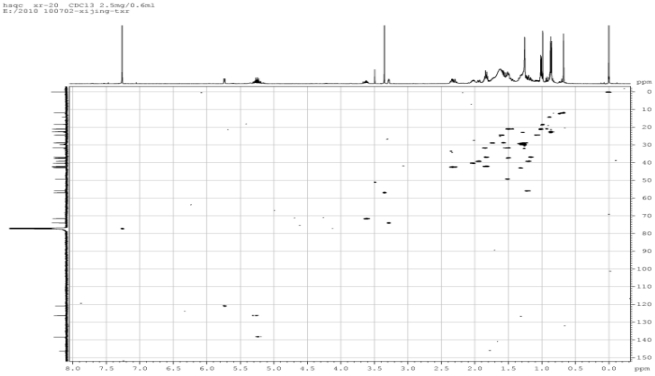
HSQC spectrum of compound **2**

**S17:**
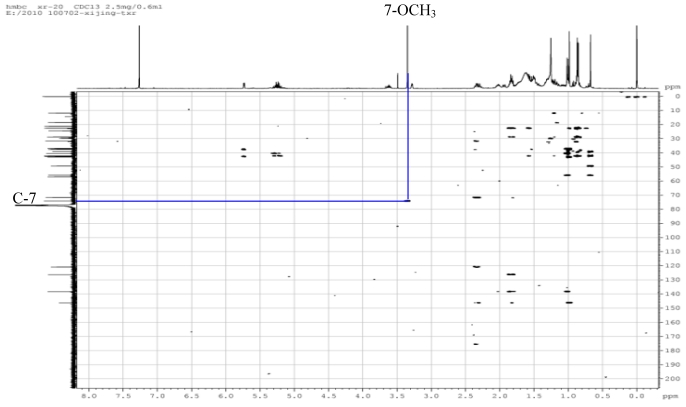
HMBC spectrum of compound **2**

**S18:**
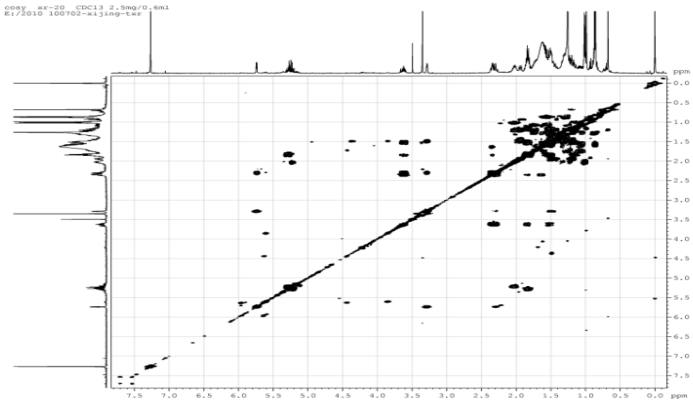
COSY spectrum of compound **2**

**S19:**
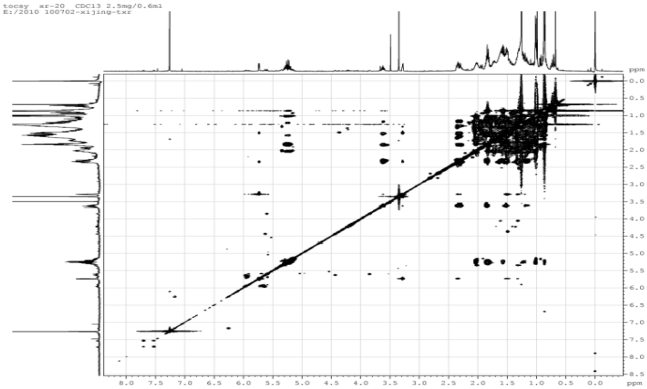
TOCSY spectrum of compound **2**

**S20:**
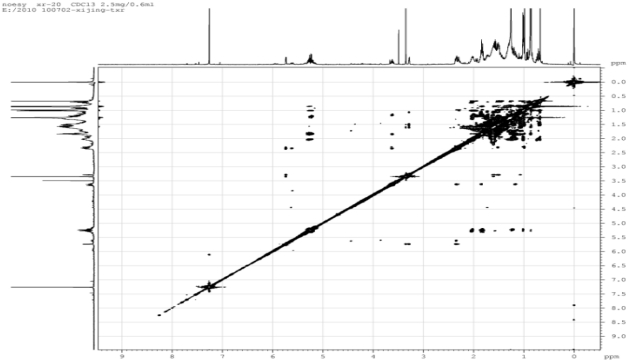
NOESY spectrum of compound **2**

## Supporting Information

S1: HR-ESI-MS of compound **1**

S2: EI-MS of compound **1**

S3: ^1^H NMR spectrum (CDCl_3_, 500 MHz) of compound **1**

S4: ^13^C NMR spectrum (CDCl_3_, 125 MHz) of compound **1**

S5: DEPT spectrum of compound **1**

S6: HSQC spectrum of compound **1**

S7: COSY spectrum of compound **1**

S8: HMBC spectrum of compound **1**

S9: TOCSY spectrum of compound **1**

S10: NOESY spectrum of compound **1**

S11: HR-ESI-MS of compound **2**

S12: EI-MS of compound **2**

S13: ^1^H NMR spectrum (CDCl_3_, 500 MHz) of compound **2**

S14: ^13^C NMR spectrum (CDCl_3_, 125 MHz) of compound **2**

S15: DEPT spectrum of compound **2**

S16: HSQC spectrum of compound **2**

S17: HMBC spectrum of compound **2**

S18: COSY spectrum of compound **2**

S19: TOCSY spectrum of compound **2**

S20: NOESY spectrum of compound **2**
